# Seismic Performance of RC Beam-Column Connections with Continuous Rectangular Spiral Transverse Reinforcements for Low Ductility Classes

**DOI:** 10.1155/2014/802605

**Published:** 2014-09-17

**Authors:** Mohammadamin Azimi, Azlan Bin Adnan, Abdul Rahman Bin Mohd Sam, Mahmood Md Tahir, Iman Faridmehr, Reza Hodjati

**Affiliations:** ^1^Faculty of Civil Engineering, Engineering Seismology and Earthquake Engineering Research (e-SEER), Department of Structure and Materials, Universiti Teknologi Malaysia (UTM), 81300 Skudai, Johor Bahru, Malaysia; ^2^Faculty of Civil Engineering, UTM Construction Research Centre (CRC), Universiti Teknologi Malaysia (UTM), 81300 Skudai, Johor Bahru, Malaysia

## Abstract

The seismic performance of RC columns could be significantly improved by continuous spiral reinforcement as a result of its adequate ductility and energy dissipation capacity. Due to post-earthquake brittle failure observations in beam-column connections, the seismic behaviour of such connections could greatly be improved by simultaneous application of this method in both beams and columns. In this study, a new proposed detail for beam to column connection introduced as “twisted opposing rectangular spiral” was experimentally and numerically investigated and its seismic performance was compared against normal rectangular spiral and conventional shear reinforcement systems. In this study, three full scale beam to column connections were first designed in conformance with Eurocode (EC2-04) for low ductility class connections and then tested by quasistatic cyclic loading recommended by ACI Building Code (ACI 318-02). Next, the experimental results were validated by numerical methods. Finally, the results revealed that the new proposed connection could improve the ultimate lateral resistance, ductility, and energy dissipation capacity.

## 1. Introduction

Incorporation of continuous spiral reinforcement in circular cross section components such as beams and columns of RC structures could improve the strength, ductility, and energy dissipation capacity of such structural members [1–3]. The first application of spiral reinforcement as the shear reinforcement to increase the axial behaviour of reinforced columns was first presented by Park in 1975 [1]. Similar studies have been performed by other scientists regarding the use of continuous spiral shear reinforcement instead of conventional stirrups, hoops, and so forth [3, 4]. In 2004, a new technique based on application of two opposing spirals (cross spirals) was reported by Mander et al. [5] where the increase in the pitch distance of spiral reinforcement did not result in any reduction in strength or ductility in column. Due to the wide application of rectangular shape cross sections in RC structures, incorporation of continuous rectangular reinforcement in RC elements has recently become more popular. Application of rectangular spiral reinforcement in RC beams was first introduced by Saatcioglu and Razvi [2] in an experimental investigation in 2005. Recently, there has been an increasing trend of studies regarding the effectiveness of rectangular spiral shear reinforcement on RC structures [6–9]. In 2011, Sheikh and Toklucu [3] conducted experiments on shear behaviour of RC T-beams reinforced by spiral-type wire ropes as internal shear reinforcements. They concluded that the maximum load bearing capacity, energy absorption, and the ductility of their connections, using the proposed method, were higher than that of conventional stirrup shear reinforcement. In addition, incorporation of rectangular continuous spiral reinforcement in RC elements could improve the seismic performance of the structural components as well [10, 11]. Once the ductility and seismic resistance of the structure is considered, ACI 318-02 recommends that continuous spiral reinforcement be used instead of conventional shear resistant one [12]. A quick review of the literature reveals the abundant amount of studies on continuous spiral reinforcement in RC elements; however, there has been little discussion about the use of rectangular spiral reinforcement as shear reinforcement in RC elements.

Since the spiral reinforcement is extended like an accordion, it could positively and quickly be tied into place. This procedure is more economical in terms of reduction of man-hours cost compared with the installation of the single closed stirrups. Moreover, formation of two end hooks for anchorage will be required for the installation of single closed stirrups. These two hooks for each closed stirrup impose extra costs on the contractors due to increased amount of steel used in their length. On the contrary, this extra length of steel will not be required in spiral reinforcement installation and hence, the total cost will be reduced as well. This benefit will become more significant in terms of RC columns where multiple stirrups per cross-section are installed plus the use of steel overlaps of stirrups [13]. Another advantage of application of spiral reinforcement would be the prevention of immature shear failure mechanism due to the continuous nature of the spirals. Yet, limited performance in torsion and questionable shear resistance in cyclic loading are listed as the weak points of both rectangular and circular spiral reinforcement.

In this study, an experimental and numerical investigation on the behaviour of low ductility class RC beam to column connections using spiral and conventional shear reinforcement systems under seismic loading, simulated by quasistatic cyclic loading, was conducted in the Laboratory of Structures and Materials, Universiti Teknologi Malaysia (UTM). Furthermore, an advanced rectangular spiral reinforcement along with a twisted opposing rectangular spiral, made of double rectangular spiral reinforcements having a pitch distance twice the pitch distance of spiral reinforcements, was also tested in this study. In the experimental portion of the study, three full scale beam to column connections were first designed in conformance with Eurocode (EC2-04) [14] for low ductility class connections and then tested by quasistatic cyclic loading recommended by ACI Building Code (ACI 318-02) [15]. In the end, the experimental results were validated with numerical results obtained from the FE Software ANSYS.

## 2. Test Program

### 2.1. Analytical Predictions

**Figure 1:**
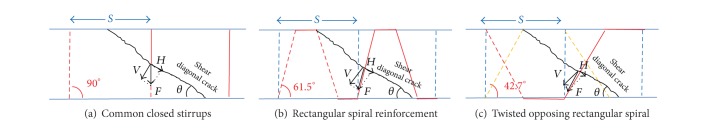
Contribution of the spiral reinforcement on the shear capacity.

The design of reinforced concrete beams is primarily based on flexural and shear strength. Once the design of a reinforced concrete member is preferred, flexure would be the first item to be considered which eventually leads to the determination of the section size and arrangement of reinforcement to provide the required resistance for moments. However, to successfully develop the plastic hinges in beams instead of columns, the beam-column relationship would be the fundamental function. Then, the beams are designed for shear failure, which is sudden with no prior warning. Hence, the shear design must be in a way so that the shear strength for every structural member exceeds the flexural strength. The mechanism of shear failure is a function of the cross-sectional dimensions, the shear reinforcement properties of the member, and type of loading. Normally, formation of the inclined shear cracks initiates at the middle height of the beam near supports at almost 45° and extends towards the compression zone. Resistance against the shear forces near supports is provided through application of anchored reinforcement that intersects these diagonal cracks. In practice, provision of shear reinforcement is done in three forms: stirrups inclined bent-up bars and a combination of a system of stirrups and bent-up bars. One great advantage of the rectangular spiral reinforcement system would be its angle that intersects the diagonal shear cracks (Figure 1). The shear design of structural members is conducted using the following equation (ACI 318-02):

(1) $$\begin{array}{c} \varphi Vn\geq Vu, \end{array}$$

where *Vu* = the factored shear force at the section, *φ* = strength reduction factor (0.85 for shear), and *Vn* = nominal shear strength computed by

(2) $$\begin{array}{c} Vn=V_{c}+V_{s}, \end{array}$$

where *V*_*c*_ = nominal shear strength provided by concrete and *V*_*s*_ = nominal shear strength provided by shear reinforcement for inclined stirrups computed by

(3) $$\begin{array}{c} V_{s}=\frac{A_{V}f_{y}d(\sin\alpha +\cos\alpha )}{S}, \end{array}$$

where *A*_*V*_ = total cross sectional area of web reinforcement within a distance *S*, for single loop stirrups and *A*_*V*_ = 2*A*_*s*_. *A*_*s*_ = cross sectional area of the stirrup bar (mm^2^). *S* = center to center spacing of shear reinforcement in a direction parallel to the longitudinal reinforcement (mm). *f*_*y*_ = yield strength of web reinforcement steel.

It can be inferred from the aforementioned equations that the shear resistance could be improved by the angle of shear reinforcement up to 1.41 times once the angle is 45°. Moreover, close installation of stirrups near the high-shear regions in conventional design would lead to congestion near the supports of the reinforced concrete beams and consequently increased time and costs. This problem could be eliminated by using rectangular spiral reinforcement which leads to improved flow of concrete within the member when it is delivered to the site by a truck mixer.

### 2.2. Case Studies and Modelling Set Up

The case studies of this research are composed of three full-scale beam to column connections using rectangular cross-section subjected to earthquake loading, simulated by quasistatic cyclic loading. The effectiveness of shear transverse patterns as a fundamental parameter was investigated in this study. The three shear transverse patterns used in this study are as follows: (i) common closed stirrups (DCL-CONVEN), (ii) rectangular spiral reinforcement (DCL-SINGLE), and (iii) twisted opposing rectangular spiral (DCL-DOUBLE). All the specimens were designed with same dimensions, with a column length 1200 mm and a beam length 600 mm, and longitudinal reinforcement designed in conformance with Eurocode (EC2-04), the Appendix, Table 3. The rebar *φ*6 was used as the transverse reinforcement, with same steel percentage in all specimens, and a distribution following the above mentioned patterns. Furthermore, to successfully isolate the low ductility class multistorey RC moment resisting frame, all specimens were considered as exterior beam to column connections. The longitudinal reinforcement of the tested beams is two longitudinal bars of diameter 8 mm as tension reinforcement and two bars of 8 mm as compression reinforcement (2*ϕ*8 top and 4*ϕ*8 bottom). The flexural tension reinforcement ratio, *ρ*_*l*_, and compression reinforcement ratio, *ρ*_*l*_′, is calculated using the following expressions and summarized in Table 1:

(4) $$\begin{array}{c} \rho_{l}=\frac{A_{sl}}{bd},\;\;\rho_{l}^{\prime }=\frac{A_{sl}^{\prime }}{bd}, \end{array}$$

where *b* and *d* are the width and the effective depth of the cross section of the beam, respectively, and *A*_*sl*_ and *A*_*sl*_′ are the area of the tension and the compression steel reinforcement, respectively. Table 1 also presents the values of the transverse reinforcement ratios, *ρ*_*t*_ and *ρ*_*tφ*_, of each beam based on the following relationships (5) and (6) for stirrups and spiral reinforcement, respectively:

(5) $$\begin{array}{c} \rho_{t}=\frac{A_{st}}{bs} \end{array}$$

(6) $$\begin{array}{c} \rho_{t\varphi }=\frac{A_{st}/2}{bs\;{\sin\varphi }_{\text{front}}}+\frac{A_{st}/2}{bs\;{\sin\varphi }_{\text{back}}}, \end{array}$$

where *A*_*st*_ = 2(*π*(*ϕ*_*t*_^2^/4)) is the area of the two-legged stirrup or the two linked spiral reinforcement with diameter *ϕ*_*t*_, *s* is the uniform spacing of the shear reinforcement, and *φ*_front_ and *φ*_back_ are the angles between the front and the back vertical link, respectively, of the spiral reinforcement and the beam axis perpendicular to the shear force.

Notice that the minimum volumetric ratio of spiral reinforcement was calculated based on following equation in accordance with ACI 318-08:

(7) $$\begin{array}{c} \rho_{h}=0.45\left(\frac{A_{g}}{A_{c}}-1\right)\frac{f_{c}^{\prime }}{f_{yh}}, \end{array}$$

where *A*_*g*_: total cross-sectional area of column section including the shell and the core. *A*_*c*_: cross-sectional area of column core confined by spiral reinforcement.

The most important criteria to evaluate RC column against seismic loading are the ratio of longitudinal steel area *A*_st_ to gross concrete cross section *A*_*g*_, *ρ*_*g*_, and transverse reinforcement index, *K*_tr⁡_. Equation (8) is used to evaluate the above mentioned parameters in accordance with ACI 318-08:

(8) $$\begin{array}{c} \rho_{g}=\frac{A_{\text{st}}}{A_{g}}\\ K_{\mathrm{tr}}=\frac{40A_{\mathrm{tr}}}{S_{\mathrm{tr}}n}, \end{array}$$

where *A*_tr⁡_ = area of transverse reinforcement within spacing *s*_tr⁡_ that crosses the splitting plane. *s*_tr⁡_ = spacing of transverse reinforcement. *n* = number of bars or wires being spliced or developed along the plane of splitting.

**Figure 2:**
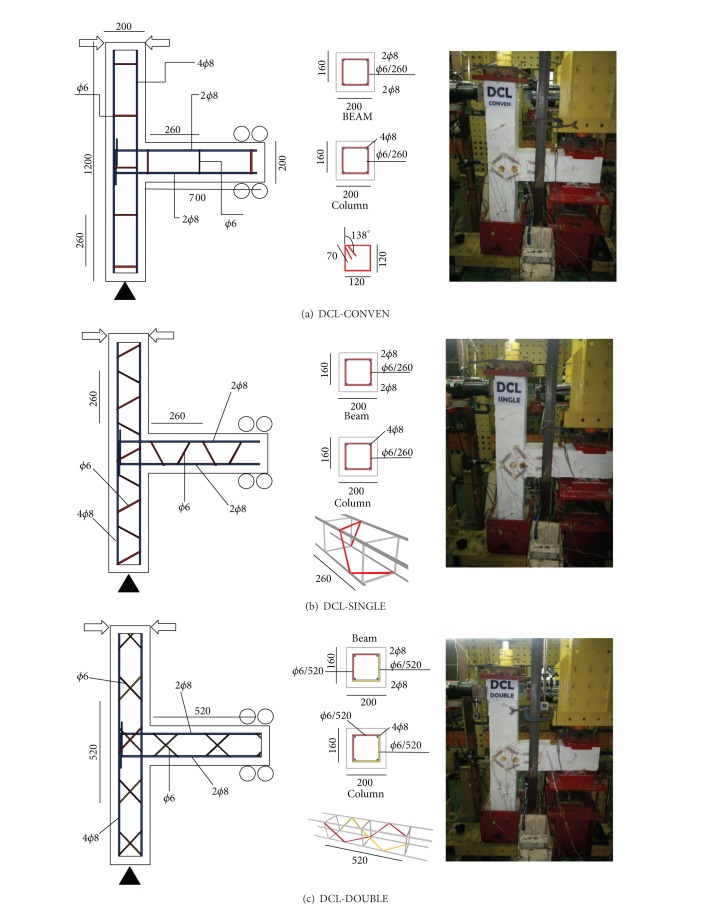
Geometry and steel reinforcement of the tested specimens, (a) DCL-CONVEN, (b) DCL-SINGLE, and (c) DCL-DOUBLE.

Notice that *K*_tr⁡_ factor shall be <2.5 imposed to avoid brittle failure and ratio of longitudinal bars should be selected between 0.01 and 0.08. The geometry and properties of the reinforcements used in this study are listed in Figure 2 and Table 1, respectively.

**Figure 3:**
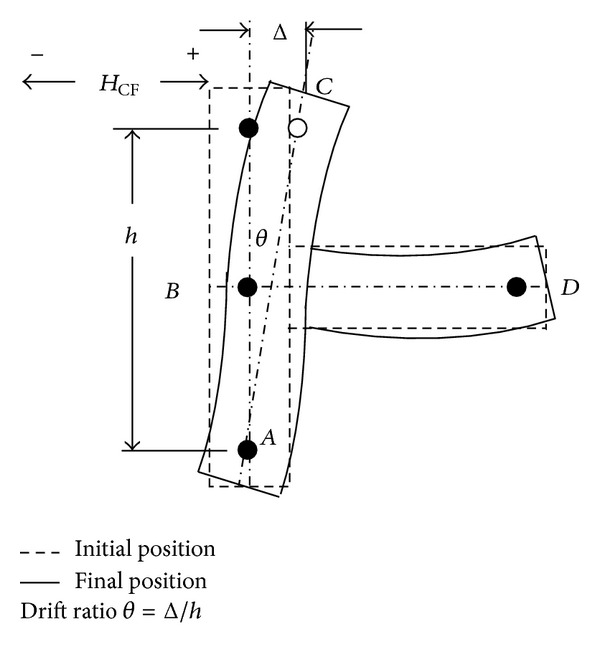
Deformation of the exterior beam to column test module [16].

A typical quasistatic cyclic pattern and modelling setup was prepared for the tests in conformance with “*Commentary on Acceptance Criteria for Moment Frames Based on Structural Testing*” (ACI T1.1R-01) [16]. Also, deformation of the exterior beam to column test module is given in Figure 3. According to the figure, the broken lines indicate the initial position of the connection considering its self-weight only. The module is pin supported at *A* and roller supported at *D*. For seismic assessment, the column is subjected to the cyclic force HCE through the pin at *C* and hence, the specimen deforms accordingly as indicated by the solid lines. The concept of drift ratio is indicated by *θ*.

**Figure 4:**
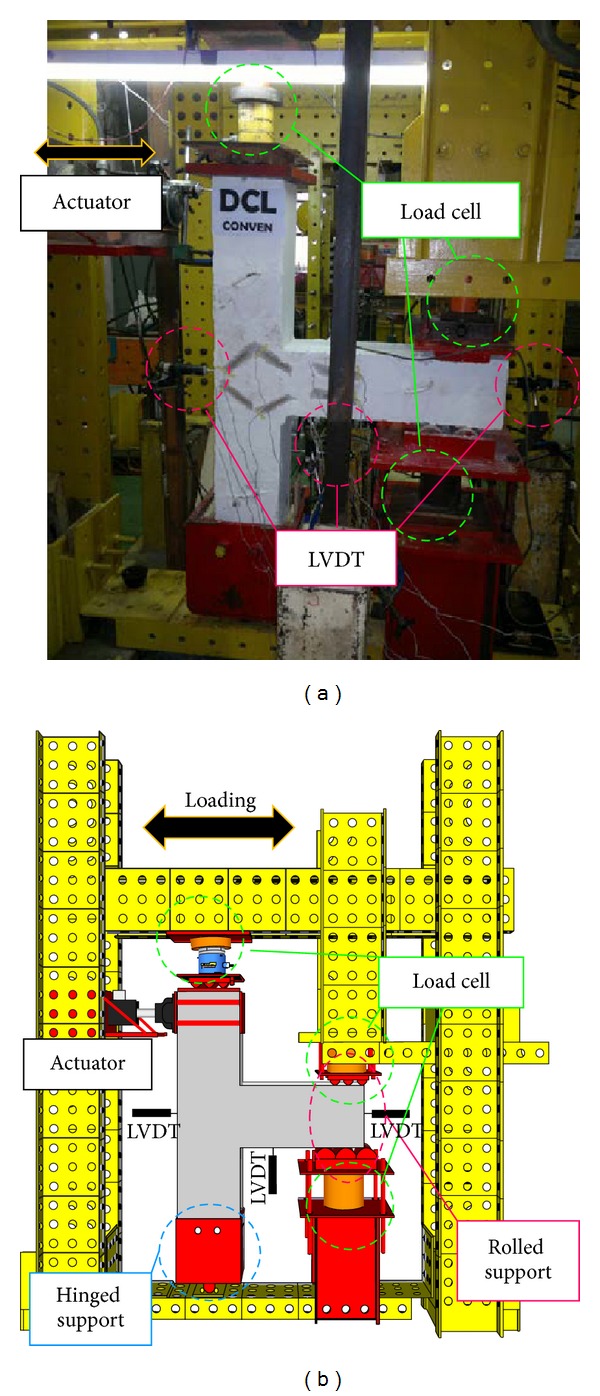
Real test setup view (a), schematic view (b).

The following statements indicate the various measurement instruments used for data collection. The DEMEC Mechanical Strain Gauge was hired as an accurate and reliable crack monitoring device at one side of specimens. Besides, TML Strain Gauges were applied to measure the degree of deformation resulting from mechanical strain. In order to record accurate strain values, the strain gauges are supposed to be correctly installed on the predicted plastic hinge locations. For the purpose of measuring the linear displacements, linear variable differential transformers (LVDT) were hired in this project; therefore, three LVDTs with accuracies of 0.01 mm were installed on the preferred locations to record the beam displacement and deflection. Also, a 250-KN hydraulic pseudodynamic actuator with a maximum piston stroke of 500 mm connected to reacting frame was used in this project. Finally, application of the axial load on top of the column, to simulate gravity, was monitored by a 50-ton load cell. The modelling setup and testing instruments are shown in Figure 4.

### 2.3. Loading Protocol

**Figure 5:**
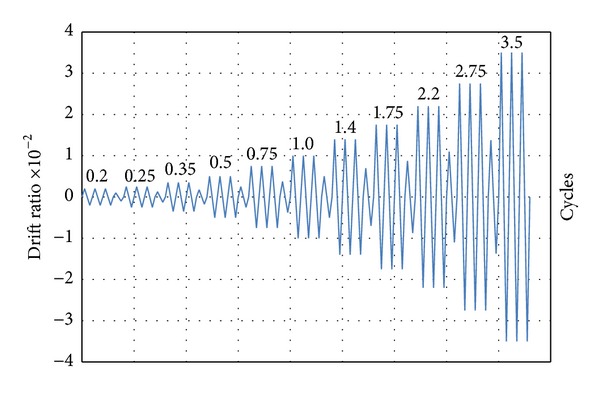
The cyclic lateral displacement pattern (the loading protocol).

The Displacement Control Method followed by the loading sequence recommended by “*Commentary on Acceptance Criteria for Moment Frames Based on Structural Testing*” (ACI T1.1R-01) provisions was used in this study. A series of load steps and the number of cycles for each one are specified in the ACI Protocol (Figure 5). Each load step corresponds to a total inter-storey drift angle. The load was incremented in a step by step manner while the data points were recorded and photographs were taken at regular intervals at the end of each load step. Once the strength of the specimens reduced to 40 percent of the maximum strength, the load steps were stopped.

The hydraulic jack and load cell were positioned vertically at the tip of the column in order to provide a constant axial compression force to the column during cyclic testing. The reason of such loading is that the effect of transverse reinforcement on the ductility of connections is significantly dependent on the axial load level. The amount of applied axial load is a function of column axial load capacity, limited to the 70% capacity to avoid the joint failure which may occur due to high compressive stresses developed in the joint core. According to finite element study increasing the column axial load level up to 30% of the column axial capacity resulted in increasing the average lateral load capacity by approximately 24%. However, in the range of 30 to 70% of the column axial capacity, no significant change in the overall behaviour was observed.

### 2.4. Materials Properties

Normal weight and ready mixed concrete with a maximum aggregate size of 20 mm were used for casting and constructing all test specimens. Casting of all specimens was performed in a horizontal layout way from the side. Then, the specimens were cured for seven days after casting in the laboratory environment. Ready mixed concrete was ordered for 28-day concrete compressive strength of 30 MP. Nevertheless, the standard cylinder test yielded a compressive strength of 35 MP. The yield strength and yield strain of the reinforcement bar used in this study were 450 MPa and 0.0022, respectively, according to the results of the Universal Test conducted in Laboratory of Structures and Materials, Universiti Teknologi Malaysia (UTM) (Table 2).

### 2.5. Numerical Study Procedure

**Figure 6:**
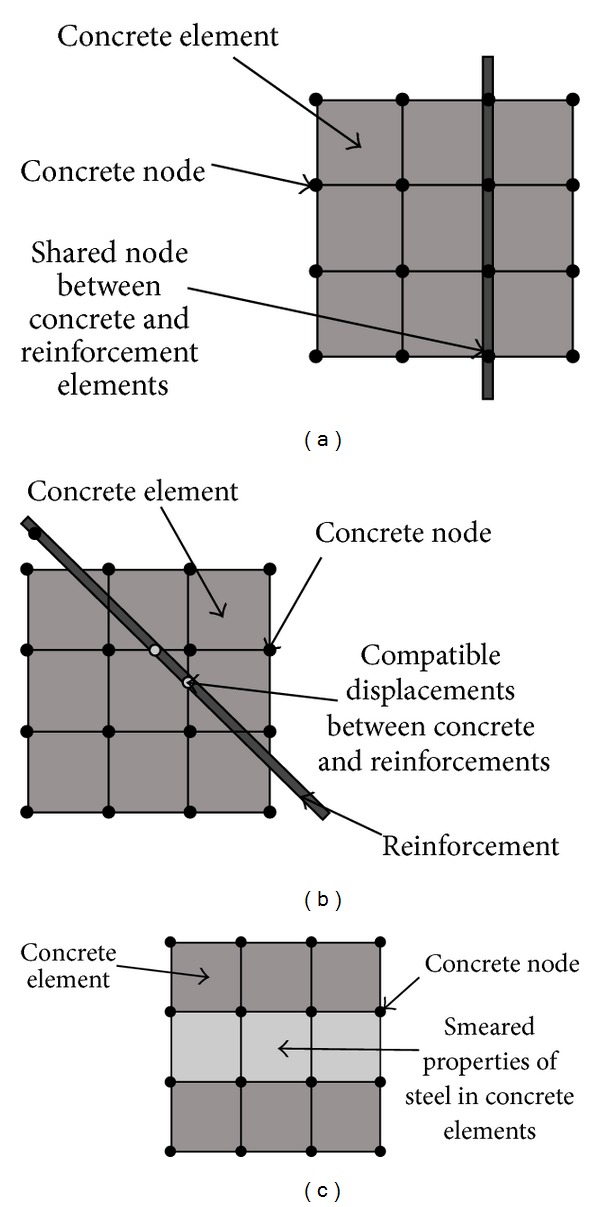
Reinforcement modelling techniques: (a) discrete, (b) embedded, and (c) smeared [4].

**Figure 7:**
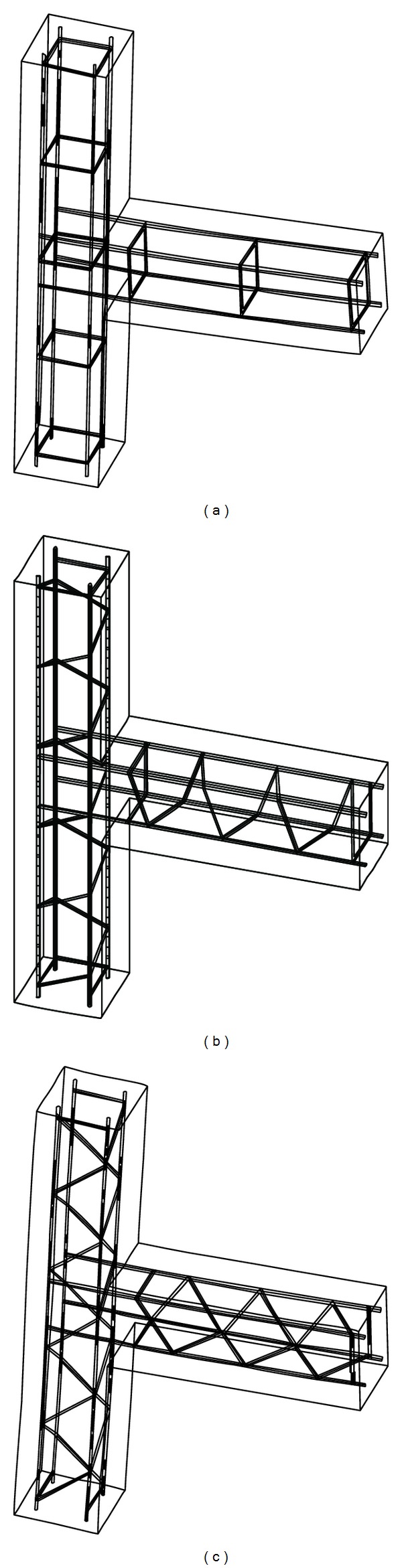
Definition of reinforcement bars with “Link180 Element” in ANSYS (a) DCL-CONVEN, (b) DCL-SINGLE, and (c) DCL-DOUBLE.

To perform the FEA phase of the study, the FE software ANSYS was used to appropriately simulate the nonlinear behaviour of beam column connections. Three-dimensional (3D) FEA was preferred to two-dimensional (2D) ones as a result of its higher accuracy. Three techniques exist in modelling the steel reinforcement in the numerical study [3–5] which as listed as (i) discrete modelling, (ii) embedded modelling, and (iii) smeared modelling (Figure 6). In this study, discrete modelling was used to model the steel bars. To efficiently describe the constitutive behaviour of the reinforcements, the isotropic strain hardening of von Mises yield criterion along with an associated flow rule were applied. The ANSYS options of “separate link 180 elements” were used to model the bars. The reinforcement modelling for all three specimens is shown in Figure 7.

**Figure 8:**
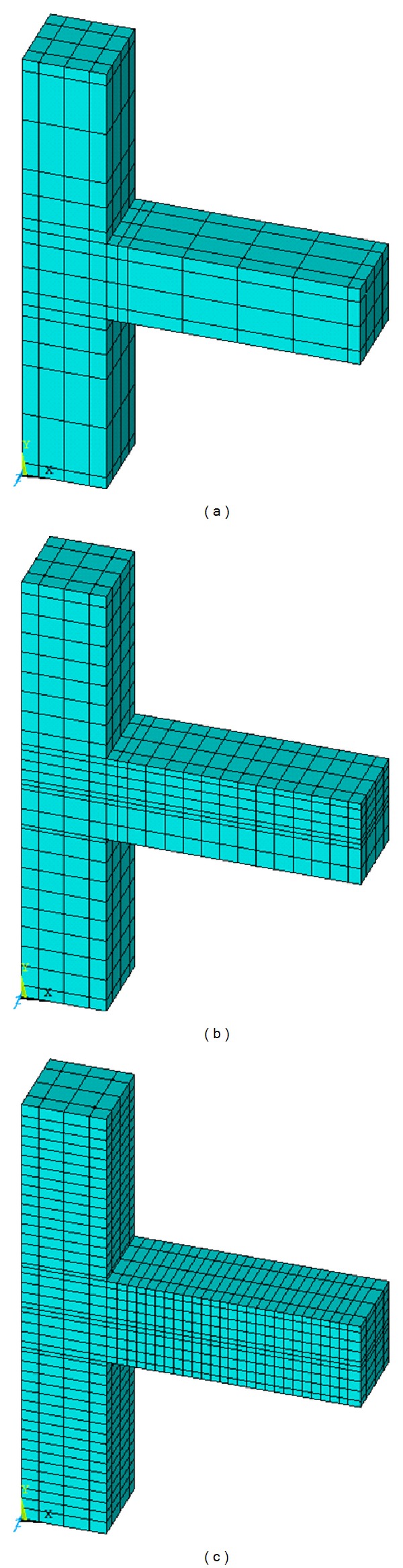
Concrete element modelling with ANSYS using the element “Solid 65” for (a) DCL-CONVEN, (b) DCL-SINGLE, and (c) DCL-DOUBLE.

To appropriately model the concrete behaviour, the “Solid65” element was hired along with application of linear isotropic and multilinear isotropic material properties. Therefore, the von Mises failure criterion with the multilinear isotropic material was used to properly define the concrete failure [17]. The concrete specimens modelled with ANSYS are shown in Figure 8.

### 2.6. Acceptance Criteria Based on ACI 318-08

According to the “Building Code Requirements for Structural Concrete” ACI 318-08, significant inelastic drift capacity must be provided by the connection through flexural yielding of the beams and limited yielding of the column and strong column-weak beam theory. Hence, an inter-storey drift angle of at least 0.035 rad must be sustained by the connection. The following requirements must be satisfied by the characteristics of the third complete cycle for cycling at drift ratio of 0.035 rad.

- The peak force must be at least 0.75 *E*_max⁡_.
- The relative energy dissipation ratio must be at least 1/8,

where *E*_max⁡_ = the maximum lateral resistance of the test specimen calculated from test results (forces or moments).

## 3. Test Results and Discussions

The test results are composed of the three following sections: (i) hysteresis responses of the specimens, (ii) energy dissipation capacity, and (iii) beam deflection and crack opening. These test results will be discussed in detail in the following paragraphs.

### 3.1. Hysteresis Responses

The fundamental parameter for investigation of seismic performance is the inter-storey drift angle. Based on the data collected by photographic documentation, data logger, and direct observation, it was concluded that initiation and propagation of cracks were observed at the same storey shear force in all specimens. However, the specimens exhibited a different performance after this point. The efficiency of different transverse shear patterns will be discussed in the following sections.

#### 3.1.1. Conventional Specimen

**Figure 9:**
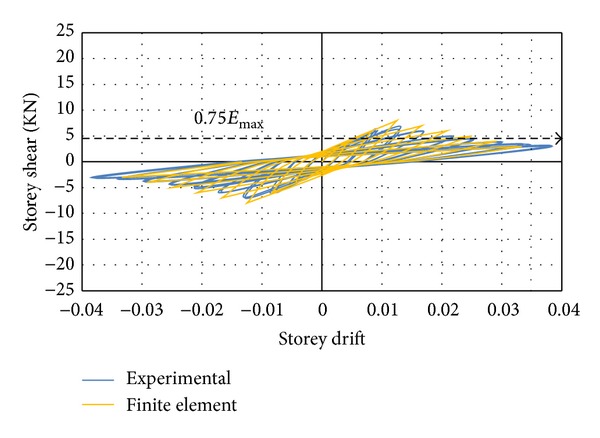
An experimental and finite element hysteresis response comparison for the DCL-CONVEN specimen.

**Figure 10:**
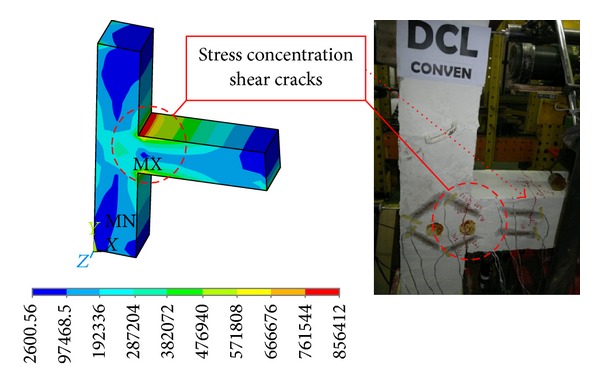
Stress intensity results and the damaged state at the end of the seismic test for the DCL-CONVEN specimen.

A poor performance was observed for specimen with conventional stirrups reinforcement subjected to cyclic loading. While the quasistatic tests were in progress, concentration of cracks was observed at the top and bottom of beam-column joint at an inter-storey drift ratio of 1.3%. No yielding plateau was indicated by the hysteresis curves while the response was brittle with an immediate increase in the storeyshear force after attaining the peak value. A severe pinching accompanied by small energy absorption was shown by the cyclic loops. The initial stiffness was reported to be higher than the stiffness at the beginning of unloading and reloading loops. There was no formation of plastic hinges in the specimen. The concrete in the joint panel governed the overall behaviour, which was an indication of the fact that the failure mode of connection, whether shear or flexural, could be identified by the amount of shear reinforcement. Therefore, the conclusion is that the failure mode could be classified as joint shear failure. Since the joint shear failure is abrupt and leads to pinched hysteresis loops with low energy dissipation, it is not desirable. The cyclic relationships between the storey shear force and the storey drift determined by numerical study and experimental test are compared in Figure 9. The damaged state of the specimen after the end of cyclic test along with stress intensity is demonstrated in Figure 10. Finally, it was concluded that a good correlation existed between the results of the numerical model and the experimental test in the overall cyclic behaviour.

#### 3.1.2. Rectangular and Twisted Opposing Rectangular Spiral Reinforcement Specimens

**Figure 11:**
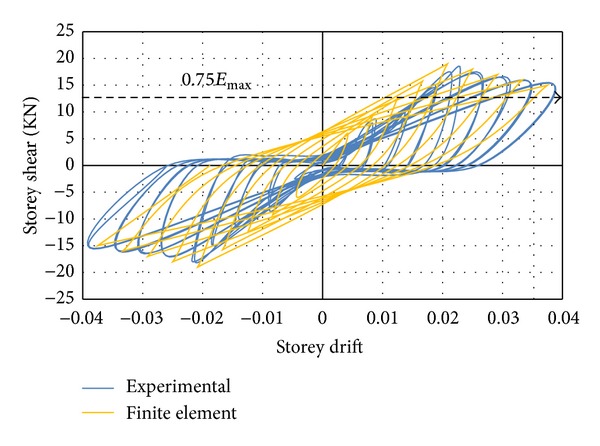
An experimental and finite element hysteresis response comparison for the DCL-SINGLE specimen.

**Figure 12:**
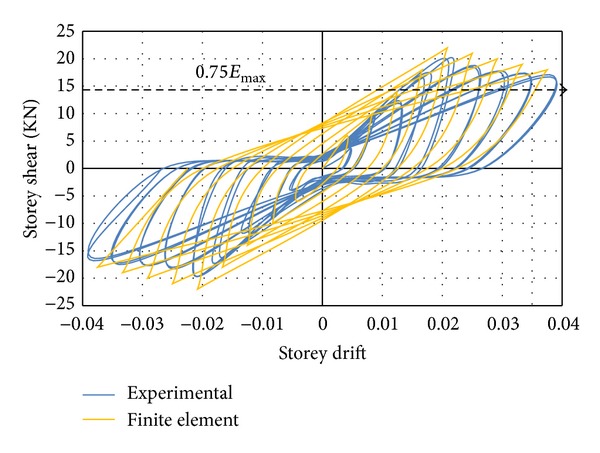
An experimental and finite element hysteresis response comparison for the DCL-DOUBLE specimen.

**Figure 13:**
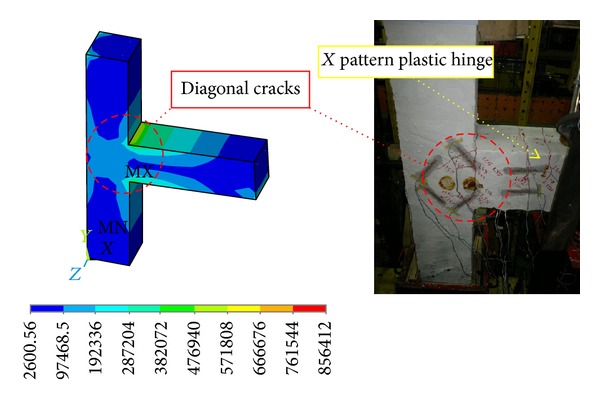
Stress intensity results and the damaged state at the end of seismic test for the DCL-SINGLE specimen.

**Figure 14:**
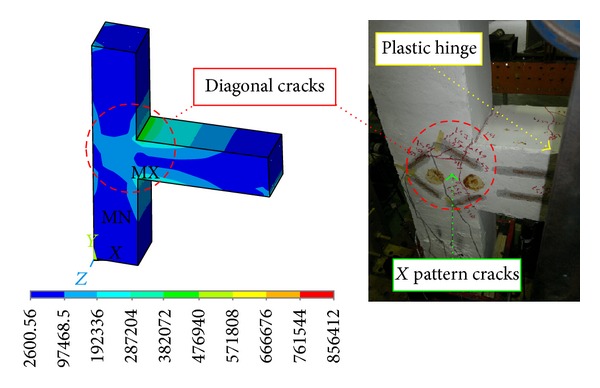
Stress intensity results and the damaged state at the end of seismic test for the DCL-DOUBLE specimen.

**Figure 15:**
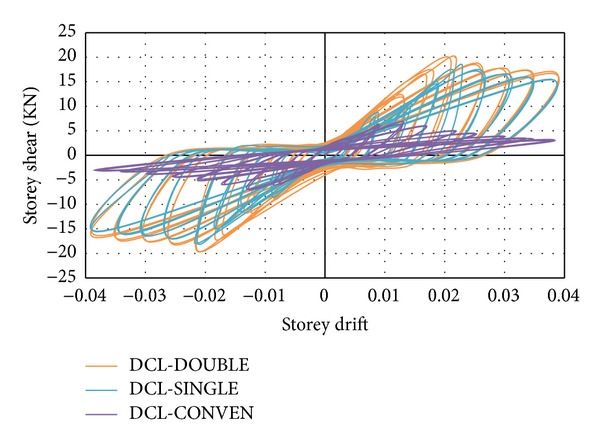
Experimental comparison of hysteresis performance of all three specimens.

Since a similar behaviour was observed by the DCL-SINGLE and DCL-DOUBLE specimens, they are discussed in the same section. Initiation of the first diagonal crack was observed in the beam at an inter-storey drift ratio of 0.5%. An X-pattern was formed by these cracks following the alternate load directions. The size and number of the diagonal cracks in joint cores kept rising until the specimen attained the peak load of almost 17.3 KN at 2.1% drift ratio for specimen DCL-SINGLE, and 19.3 KN at 2.1% drift ratio for specimen DCL-DOUBLE. After this cycle, there was no crack observation in the beam; however, diagonal cracks continued to widen in the joint core followed by spalling of concrete at the center of the joint area. Extension of concrete spalling throughout a wider joint area, exposing column longitudinal bars occurred at 2.5% drift. The 4800 *με* (microstrain) value recorded by the strain gauges positioned on appropriate locations was a sign of strength degradation resulting from yielding of longitudinal bars which was relevant to the 3% top drift in the experimental test. The failure of both connections was categorized as ductile flexural failure. The wide hysteresis loops were an indication of large energy dissipation in bending mode. An acceptable correspondence behaviour is observed between the experimental results and numerical analysis of the specimens, where this reasonable correlation is highlighted in Figures 11 and 12. Besides, the experimental failure mode and the equivalent strain distribution at the end of cyclic test are highlighted in Figures 13 and 14. Finally, an experimental comparison between hysteresis performances of all specimens is shown in Figure 15.

### 3.2. Energy Dissipation Capacity and Load-Drift Envelops

**Figure 16:**
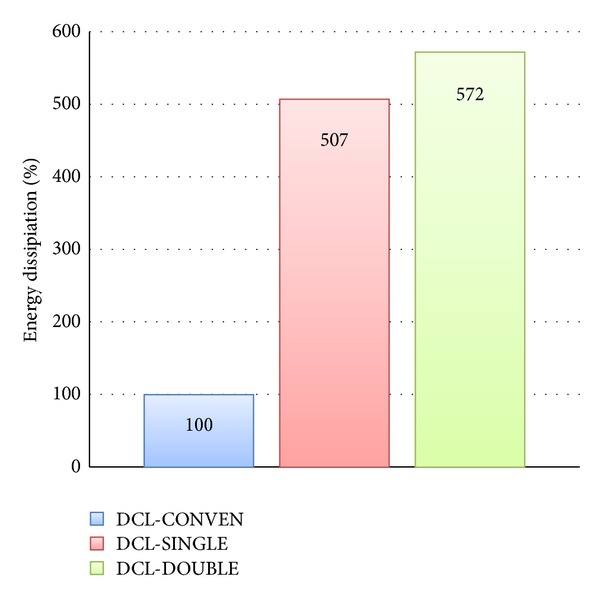
An energy dissipation capacity comparison for the DCL group.

The fundamental parameter in resisting seismic loading is known to be the energy dissipation capacity. In this study, energy dissipation of connections in each group was determined using the area enclosed by the lateral load-displacement loops. A comparison between the energy dissipation capacities of all three connections is depicted in Figure 16. It is evident from the bar graph that a better performance in absorbing the cyclic energy was demonstrated by the rectangular spiral reinforcement (DCL-SINGLE) and twisted opposing rectangular spiral (DCL-DOUBLE) compared to the common closed stirrups (DCL-CONVEN).

**Figure 17:**
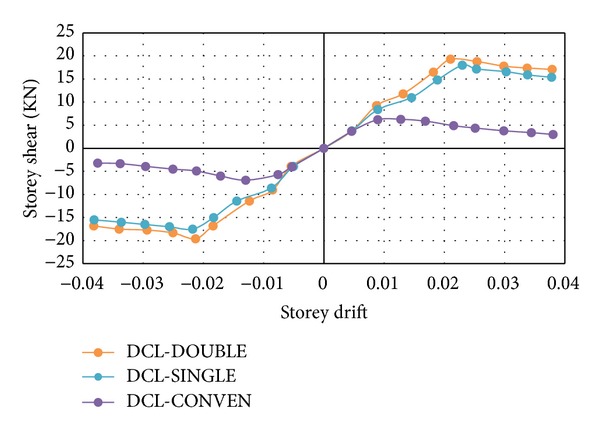
Envelop curves for comparing the maximum load in each cycle for the DCL group.

The shear deformation of beam to column connection makes a great contribution to envelope curves in RC frames. The nonlinear behaviour of the tested beam-column joints is reflected in the envelope curves. The peak lateral resistance values calculated at each level of drift and the corresponding drift ratios were incorporated to draw the envelope curves. According to Figure 17, the DCL-SINGLE and DCL-DOUBLE curves are capable of withstanding large drifts compared to DCL-CONVEN due to the impact of the special shear transverse patterns of the DCL-SINGLE and DCL-DOUBLE specimens. Also, it is evident that the peak lateral resistances of DCL-SINGLE and DCL-DOUBLE specimens are almost similar.

### 3.3. Beam Deflection and Crack Opening

**Figure 18:**
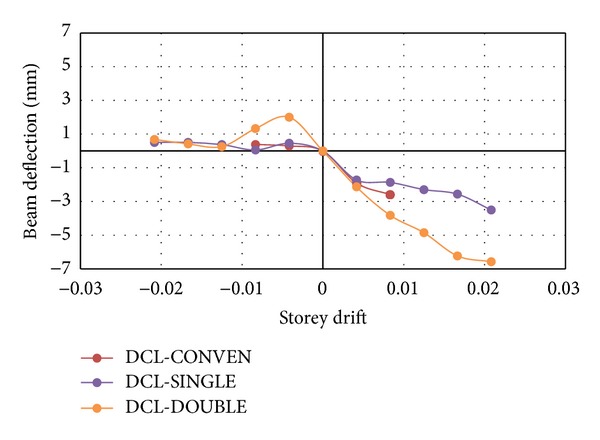
The drift versus beam deflection curves for both loading directions, positive and negative for the DCL group.

The overall deflection of the specimens was a function of their shear characteristics. A higher deflection was observed for the DCL-SINGLE and DCL-DOUBLE specimens leading to higher impacts and higher absorption of energy. It is good to mention that deflection measurement was conducted through installation of a vertical LVDT at the bottom of the beams. However, minor deflections at the ultimate load along with a relatively brittle failure were observed for the DCL-CONEN specimen. It can be inferred from the results that the deflections and modes of failure of the connections are a function of the appropriate pattern and amount of shear reinforcement. The drift versus deflection curves for all specimens is shown in Figure 18.

**Figure 19:**
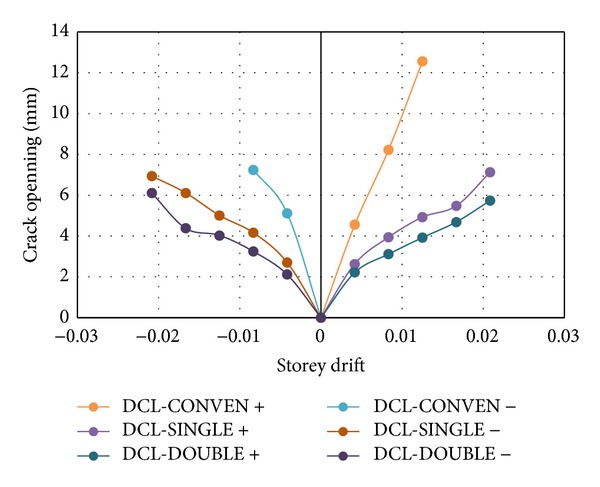
Crack opening curves for the DCL group in the critical zone for both loading directions, positive and negative for the DCL group.

Crack opening in RC members is usually accompanied by shear crack sliding along shear cracks leading to shear transfer by the aggregate interlock mechanism. Shear sliding, which is related to shear opening, is the key factor in fracturing shear reinforcement, particularly under cyclic loading. Moreover, the angle between the shear reinforcement and shear cracks significantly affected the diagonal crack openings. Nevertheless, the beam with vertical stirrups showed greater shear crack widths. The crack opening curves for all specimens are shown in Figure 19.

## 4. Summary and Conclusive Remarks

The seismic performance of a new proposed beam to column connection introduced as “twisted opposing rectangular spiral (DCL-DOUBLE)” was experimentally and numerically compared against rectangular spiral (DCL-SINGLE) and conventional (DCL-CONVEN) shear reinforcement systems in this study. The fundamental acceptance criteria selected for seismic assessment of the connections in this study was in conformance with ACI 318-08. The main findings of this research based on the numerical and experimental results are listed as follows.

- The failure modes of RC beam to column connections, whether ductile flexural failure or joint shear failure, were affected by the angle between the shear reinforcement and shear cracks. Hence, a higher capacity of connected beam was developed by the DCL-DOUBLE and DCL-SINGLE specimens compared to that of the conventional (DCL-CONVEN) shear reinforcement system.
- A 3.5% drift angle was achieved by both the DCL-DOUBLE and DCL-SINGLE specimens with relative energy dissipation not lesser than 1/8. In other words, although the original design of the rectangular spiral reinforcement specimens was nonseismic, they eventually met the seismic requirements.
- A higher energy dissipation capacity was demonstrated by the DCL-DOUBLE specimen compared to that of the DCL-SINGLE one. This higher energy dissipation capacity is attributed to the existence of double spiral transverse reinforcements in each section of the beam to column connection, which resist efficiency to cyclic loading.
- The shear transverse pattern significantly influenced the overall deflection and crack opening of the specimens. A significant deflection will be provided by the rectangular spiral reinforcement which results in higher ductility. Furthermore, a widespread crack distribution was observed for both the DCL-DOUBLE and DCL-SINGLE specimens compared to that of the DCL-CONVEN one.

## Figures and Tables

**Table 1:** Reinforcement details of specimens.

Specimen class	Beam reinforcement						Column reinforcement	
	*ρ*_*l*_ (%)	*ρ*_*l*_′ (%)	*ρ*_*t*_ (%)	*ρ*_*tφ*_ (%)	*φ*_front_ (deg)	*φ*_back_ (deg)	*ρ*_*g*_ (%)	*K*_tr⁡_
DCL-CONVEN	0.359	0.359	0.108	—	—	—	1.41	4.349
DCL-SINGLE	0.359	0.359	—	0.117	68	112	1.41	4.349
DCL-DOUBLE	0.359	0.359	—	0.139	51	129	1.41	4.349

**Table 2:** Mechanical properties of reinforcing bars.

Bar size	Bar diameter (mm)	Bar area (mm^2^)	Modulus of elasticity (GPa)	Yield strength (MP)	Yield strain mm/mm
*φ*8	8.5	60	200	450	0.0022
*φ*6	6.2	30	200	450	0.0022

**Table 3:** Connection design formulas for ductility classes low in Eurocode.

DCL		
Beam	**Longitudinal Bars (L)**	
	**Critical Region Length **	*h*_*w*_
	*ρ*_min⁡_	0.5*f*_ctm_/*f*_yk_, 0.13%
	*ρ*_max⁡_	0.04
	**Transverse bars (w)**	
	**Outside Critical Regions**	
	Spacing *s*_*w*_ ≤	0.75 d
	*ρ*_*w*_ ≥	0.08(*f*_ck_)^1/2^/*f*_yk_
	**In Critical Regions**	
	Spacing *s*_*w*_ ≤	—
	*d*_bw_ ≥	6 mm

Column	**Longitudinal Bars (L)**	
	**Critical Region Length**	—
	*ρ*_min⁡_	0.1*N*_*d*_/*A*_*c*_ *f*_yd_, 0.2%
	*ρ*_max⁡_	4%
	Bars per side	**2**
	Spacing between restrained bars	—
	**Transverse bars (w)**	
	**Outside Critical Regions**	
	*d*_bw_ ≥	6 mm, *d*_bl_/4
	Spacing *s*_*w*_ ≤	20*d*_bl_, min⁡(*h*_*c*_, *b*_*c*_), 400 mm
	**In Critical Regions**	
	*d*_bw_ ≥	6 mm, *d*_bl_/4
	Spacing *s*_*w*_ ≤	—

where *f*_ck_ is characteristic compressive cylinder strength of concrete at 28 days, *f*_cd_ is design value of concrete compressive strength, *f*_ctm_ is mean value of axial tensile strength of concrete, *f*_yk_ is characteristic yield strength of reinforcement, *f*_yd_ is design yield strength of reinforcement, *f*_ywd_ is design yield of shear reinforcement, d is effective depth of section, *d*_bl_ is longitudinal bar diameter, *d*_bw_ is diameter of hoop, *h*_*w*_ is cross-sectional depth of beam, *h*_*c*_ is cross-sectional depth of column in the direction of interest, *ε*_sy,*d*_ is design value of steel strain at yield, *μ*_*∅*_ is curvature ductility factor, *ρ*_*w*_ is shear reinforcement ratio, *ρ*′ is compression steel ratio in beams, and *b*_0_ is width (minimum dimension) of confined concrete core (to centreline of hoops).

## Appendix

See Table 3.
